# Determinants of Slow Walking Speed in Ambulatory Patients Undergoing Maintenance Hemodialysis

**DOI:** 10.1371/journal.pone.0151037

**Published:** 2016-03-28

**Authors:** Yoshifumi Abe, Atsuhiko Matsunaga, Ryota Matsuzawa, Toshiki Kutsuna, Shuhei Yamamoto, Kei Yoneki, Manae Harada, Ryoma Ishikawa, Takaaki Watanabe, Atsushi Yoshida

**Affiliations:** 1 Department of Rehabilitation Sciences, Kitasato University Graduate School of Medical Sciences, Sagamihara, Kanagawa, Japan; 2 Department of Rehabilitation, Kitasato University Hospital, Sagamihara, Kanagawa, Japan; 3 Department of Rehabilitation, Kitasato University East Hospital, Sagamihara, Kanagawa, Japan; 4 Hemodialysis Center, Sagami Junkanki Clinic, Sagamihara, Kanagawa, Japan; Vanderbilt University, UNITED STATES

## Abstract

Walking ability is significantly lower in hemodialysis patients compared to healthy people. Decreased walking ability characterized by slow walking speed is associated with adverse clinical events, but determinants of decreased walking speed in hemodialysis patients are unknown. The purpose of this study was to identify factors associated with slow walking speed in ambulatory hemodialysis patients. Subjects were 122 outpatients (64 men, 58 women; mean age, 68 years) undergoing hemodialysis. Clinical characteristics including comorbidities, motor function (strength, flexibility, and balance), and maximum walking speed (MWS) were measured and compared across sex-specific tertiles of MWS. Univariate and multivariate logistic regression analyses were performed to examine whether clinical characteristics and motor function could discriminate between the lowest, middle, and highest tertiles of MWS. Significant and common factors that discriminated the lowest and highest tertiles of MWS from other categories were presence of cardiac disease (lowest: odds ratio [OR] = 3.33, 95% confidence interval [CI] = 1.26–8.83, P<0.05; highest: OR = 2.84, 95% CI = 1.18–6.84, P<0.05), leg strength (OR = 0.62, 95% CI = 0.40–0.95, P<0.05; OR = 0.57, 95% CI = 0.39–0.82, P<0.01), and standing balance (OR = 0.76, 95% CI = 0.63–0.92, P<0.01; OR = 0.81, 95% CI = 0.68–0.97, P<0.05). History of fracture (OR = 3.35, 95% CI = 1.08–10.38; P<0.05) was a significant factor only in the lowest tertile. Cardiac disease, history of fracture, decreased leg strength, and poor standing balance were independently associated with slow walking speed in ambulatory hemodialysis patients. These findings provide useful data for planning effective therapeutic regimens to prevent decreases in walking ability in ambulatory hemodialysis patients.

## Introduction

Recent studies have shown that walking ability is a powerful predictor of life expectancy among older adults [1–4]. In particular, reduced walking ability characterized by slow walking speed is independently associated with mortality, hospitalization, and adverse events in healthy older individuals and those with heart disease, kidney disease, stroke, and other chronic diseases in epidemiological cohort studies [5–9]. Therefore, assessment and management of risk factors related to slow walking speed may be necessary to implement effective disease management and secondary interventions for patients with chronic diseases.

Ambulatory patients with end-stage renal disease undergoing dialysis have a decreased walking speed, at approximately 60% of that of age-matched norms [10]. Our previous studies also showed that a lower level of habitual physical activity is an independent risk factor for all-cause mortality, and is closely associated with walking speed in maintenance hemodialysis patients [11, 12]. Thus, an intervention strategy aimed at maintaining and improving walking ability may be important in implementing an effective disease management program for hemodialysis patients. However, very few studies have examined factors associated with slow walking speed in this population. Moreover, maintenance hemodialysis patients often have several complications and comorbidities, including diabetes, orthopedic abnormality, peripheral neuropathy, and peripheral artery disease, which directly or indirectly cause deterioration in walking ability [13–17]. These underlying conditions complicate the identification of determinants of slow walking speed.

This study aimed to identify factors associated with slow walking speed in clinically stable ambulatory patients undergoing hemodialysis by conducting multivariate analysis adjusted for the complications.

## Materials and Methods

This study was approved by the Ethics Committee of Kitasato University School of Allied Health Sciences (2012–020), and we obtained written informed consent from all participants after the school protocol was explained in detail. This study was conducted in accordance with the standards set forth by the latest revision of the Declaration of Helsinki. Between October 2012 and March 2014, there were 324 Japanese outpatients undergoing maintenance hemodialysis three times per week at the Hemodialysis Center at Sagami Junkanki Clinic. We recruited patients who did not exercise. Subjects of this cross-sectional study were recruited from these patients, based on the following exclusion criteria: the duration of maintenance hemodialysis ≤3 months; hospitalization ≤3 months prior to study enrollment; history of recent myocardial infarction or angina pectoris; presence of uncontrolled cardiac arrhythmias, hemodynamic instability, uncontrolled hypertension, or renal osteodystrophy with severe arthralgia; history of fracture in the lower extremities or spine ≤1 year prior to study enrollment; requirement for walking assistance; and other conditions that limited walking (e.g., dementia, low vision or blindness, paralysis due to stroke, leg amputation).

### Clinical characteristics

Information regarding age, sex, height, weight, body mass index (BMI), hemodialysis duration, primary cause of end-stage renal disease, blood hemoglobin, and serum albumin concentration was obtained from clinical records. BMI was calculated by dividing weight in kilograms by the square of height in meters. Blood hemoglobin and serum albumin concentrations were measured immediately before each hemodialysis session.

The presence of comorbidities (i.e., diabetes mellitus, peripheral arterial disease, cardiac disease, cerebrovascular disease, peripheral neuropathy, and orthopedic abnormality), and the number of comorbidities if present, were assessed based on clinical records. Peripheral arterial disease was diagnosed as skin perfusion pressure at the dorsum of foot lower than 50 mmHg using a Laser Doppler probe (PAD3000; Kaneka, Osaka) [18–20]. Patients were defined as having heart disease if they had a history of angina pectoris, myocardial infarction, percutaneous coronary intervention, or coronary artery bypass grafting, and/or had evidence of hemodynamically significant valvular disease by echocardiography or cardiac catheterization. Patients were defined as having cerebrovascular disease if they had a history of stroke confirmed by brain imaging (computed tomography or magnetic resonance imaging), or presented with focal signs or symptoms that were consistent with stroke. Peripheral neuropathy was diagnosed by clinical tests of ankle reflex, vibration perception, and pressure sensation, which are commonly employed as screening tests for diabetic neuropathy [21–25]. The ankle reflex test was conducted in either the kneeling position or resting position on a couch using a tendon hammer, and was defined as abnormal when the Achilles tendon reflex was absent either at rest or upon reinforcement. The vibration perception test was performed using a timed method with a 128-Hz tuning fork; patients were asked to keep their eyes closed during the test, and to inform the examiner when they perceived the vibration on the medial malleolus of the ankle joint to have stopped. Perception of vibration ≤10 seconds was defined as abnormal perception. The pressure sensation test was performed using a 10-g monofilament (5.07 Semmes-Weinstein). Patients responded “yes” or “no” when asked whether the monofilament was being applied to a particular site, with their eyes closed. Those with incorrect answers to two out of three applications were defined as having abnormal sensation. Patients were determined to have peripheral neuropathy if they had at least one abnormality in the above-mentioned three tests. Orthopedic abnormality was defined as having radiographic evidence of advanced (mild to severe) osteoarthritis of the spine, hip, knee, or ankle, a prior history of total hip or knee arthroplasty, or a history of fracture in the spine or lower extremities.

### Walking ability

Self-selected maximum walking speed (MWS) along a 10-meter walkway was measured to assess patient walking ability. The validity and reliability of the 10-meter walk test, which measures the time (in seconds or minutes) required for a patient to walk 10 m, have been investigated in several populations, from healthy elderly to patients with stroke, neurological disorders, and orthopedic dysfunction [12,26,27]. MWS was measured twice at an interval of 30 s while the patient walked a distance of 10 m at the maximum speed without running. The highest values for each walking speed, expressed in meter per minutes, were represented as the walking ability.

### Motor function

Assessment of motor function included measurements of maximum leg strength, lower extremity flexibility, and standing balance. Maximum leg strength was evaluated using a hand-held dynamometer (μtas F-1; Anima, Tokyo, Japan). Patients were asked to sit on a bench with their hip and knee flexed at an angle of 90 degrees, and then the maximum voluntary isometric knee extensor strength was measured three times. Maximum leg strength was expressed as a percentage of body weight, i.e., the average of right and left maximum isometric leg strength divided by weight [12, 27].

The passive range of motion was measured for knee, ankle, and great toe metatarsophalangeal joints to assess lower extremity flexibility according to guidelines for the range of motion test [28]. Lower extremity flexibility was defined as the sum of the extension angle of the knee joint, dorsiflexion angle of the ankle joint, and extension angle of the great toe metatarsophalangeal joint.

Standing balance was evaluated by measuring one-leg standing time, which reflects the ability to maintain the center of pressure within the base of support of the body. The duration of time that subjects can stand on one leg with their eyes open, while holding their hands on their waist without any aid or falling, was measured using a stopwatch. The measurement was stopped if subjects hopped, stepped, put the raised foot down on the other foot or on the floor, or released their hands from the waist to balance. Subjects underwent a second trial if they were unable to stand on one leg for 60 seconds in the first trial [29].

### Statistical analysis

As there are no established walking speed categories for hemodialysis patients, MWS was categorized into sex-specific tertiles based on previous reports that a strong association exists between MWS and sex [30]. To maintain a balanced sample size in men and women, cutoffs were defined by sex as follows: lowest tertile (i.e., slow walking speed), <88.8 m/min of MWS in men and <80.4 m/min of MWS in women; middle tertile (i.e., intermediate walking speed), 88.8–106.2 m/min of MWS in men and 80.4–98.4 m/min of MWS in women; and highest tertile (i.e., fast walking speed), ≥106.2 m/min of MWS in men and ≥98.4 m/min of MWS in women.

MWS data for all patients were assigned by sex using a histogram of speed intervals (10 m/min). Differences in clinical characteristics (age, sex, height, weight, BMI, hemodialysis duration, primary cause of end-stage renal disease, blood hemoglobin, serum albumin concentration, and conditions and number of comorbidities), walking speed, and motor function across tertiles of walking speed were assessed for significance using one-way analysis of variance (ANOVA) and the χ^2^-test. Univariate and stepwise multivariate logistic regression analyses were performed to assess whether clinical characteristics and motor function could discriminate the lowest tertile from the middle and highest tertiles, and the lowest and middle tertiles from the highest tertile.

All analyses were performed using the Statistical Package for Social Sciences (IBM SPSS Statistics 21.0 for Mac; IBM Corp., Armonk, NY, USA). Statistical significance was set as *P* < 0.05.

## Results

### Patient characteristics, maximum walking speed, and motor function

**Fig 1:**
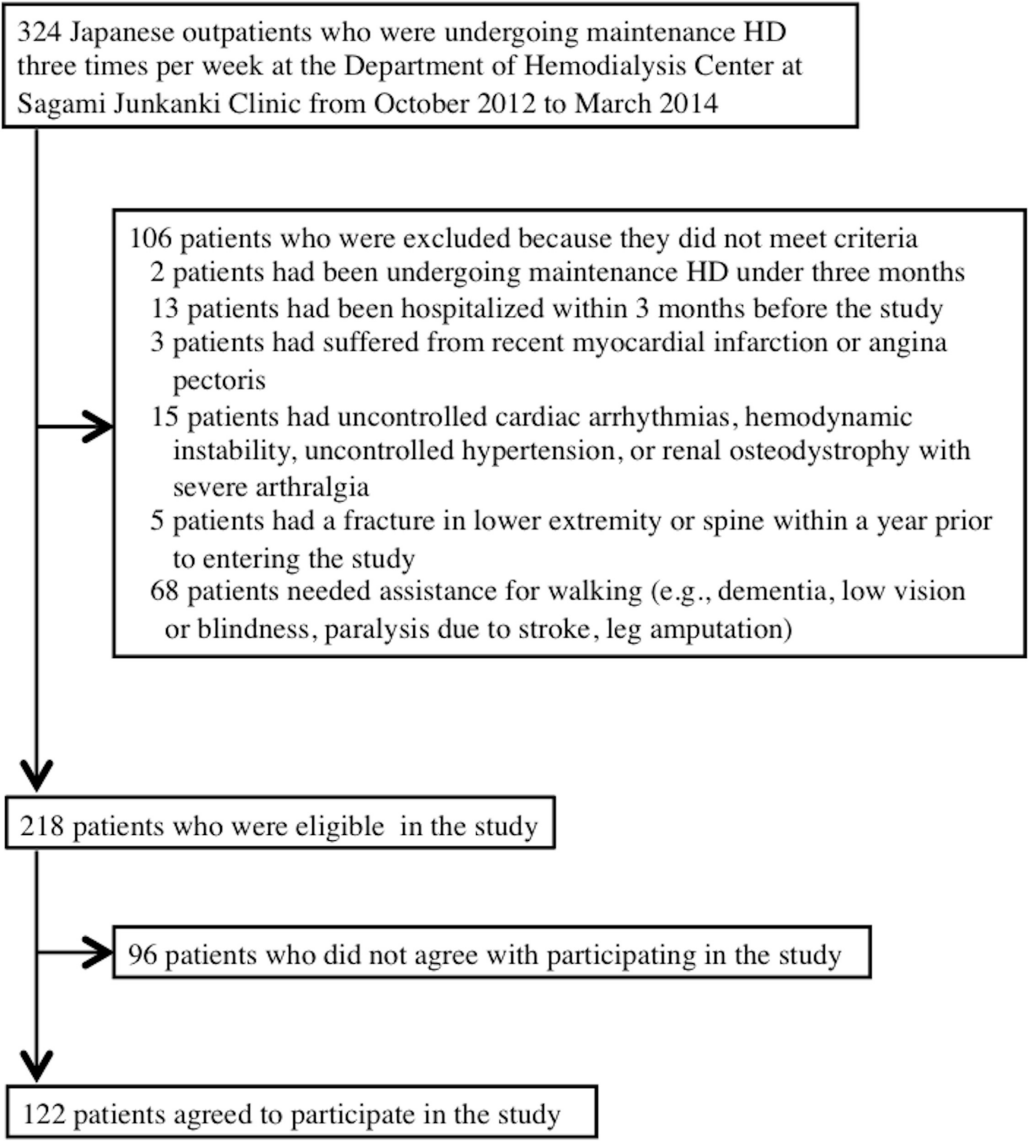
Flow diagram of the participant selection and exclusion process.

As shown in Fig 1, 106 of the 324 Japanese hemodialysis outpatients who were assessed for eligibility fell under the exclusion criteria, and 96 declined to participate. Consequently, 122 patients (64 men, 58 women) were included in this study.

**Fig 2:**
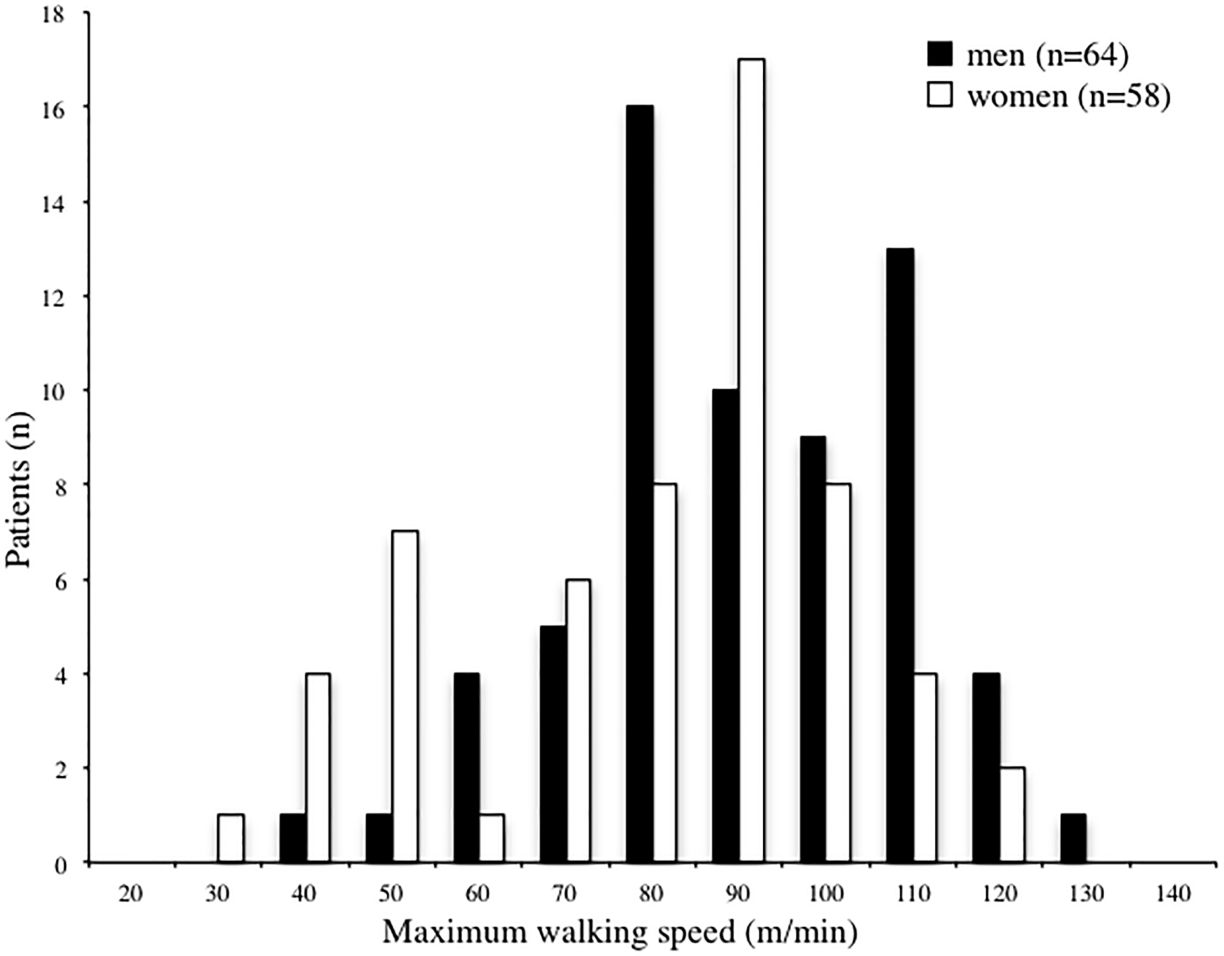
Histogram of maximum walking speed. Maximum walking speed was calculated in meters per minute. Open and closed bars indicate women and men, respectively.

Table 1 shows the clinical characteristics, MWS, and motor function of all 122 patients (mean age, 68 years; age range, 42–93 years). The most common underlying kidney disease was diabetic nephropathy (34.7%), followed by glomerulonephritis (28.8%). Mean MWS was 91.2 ± 20.4 m/min, and the distribution of walking speed by sex is shown in a histogram in Fig 2 Median MWS in men and women were 95.1 m/min and 92.5 m/min, respectively.

**Table 1:** Patient characteristics, walking speed, and motor function.

Age (years)	68 (± 9)
Men (%)	52.5
Height (cm)	158.8 (± 8.0)
Weight (kg)	54.0 (± 10.2)
BMI (kg / m^2^)	21.2 (± 3.7)
HD duration (years)	8.6 (± 8.6)
Primary cause of end-stage renal disease (%)	
Glomerulonephritis	28.8
Diabetic nephropathy	34.7
IgA nephropathy	7.6
Polycystic kidney	3.4
Nephrosclerosis	4.2
Unknown	13.6
Others	7.6
Laboratory values	
Hemoglobin (g / dL)	11.8 (± 4.2)
Serum albumin (g / dL)	3.8 (± 0.3)
Comorbid conditions	
Diabetes mellitus (%)	43.4
Peripheral arterial disease (%)	44.9
Cardiac disease (%)	57.4
Cerebrovascular disease (%)	22.1
Peripheral neuropathy (%)	30.5
Orthopedic abnormality (%)	61.5
Joint disease (%)	42.6
History of fracture (%)	18.9
Others (%)	29.5
The number of comorbidities	2.6 (± 1.3)
Walking ability	
Maximum walking speed (m/min)	91.2 (± 20.4)
Motor function	
Leg strength (%BW)	42.0 (± 13.0)
Lower extremity flexibility (degrees)	187.0 (± 32.3)
Standing balance (seconds)	29.8 (± 25.6)

Date are presented as mean (± standard deviation) or number of subject. BMI: body mass index, HD: hemodialysis, BW: body weight.

Differences in clinical characteristics, MWS, and motor function across tertiles of walking speed are shown in Table 2. Significant differences across tertiles of walking speed were found in age (P<0.05), presence of cardiac disease (P<0.05) and peripheral neuropathy (P<0.05), history of fracture (P<0.01), number of comorbidities (P<0.001), MWS (P<0.001), leg strength (P<0.001), and standing balance (P<0.001).

**Table 2:** Differences in the clinical characteristics, maximum walking speed, and motor function across tertiles of maximum walking speed.

	Highest tertile (N = 40)	Middle tertile (N = 40)	Lowest tertile (N = 42)	P value
Age (years)	66 (± 8)	67 (± 8)	71 (± 10)	0.03
Men (%)	55.0	52.5	50.0	0.90
Height (cm)	160.0 (± 7.2)	159.7 (± 8.7)	157.2 (± 8.2)	0.23
Weight (kg)	54.3 (± 9.1)	54.7 (± 10.6)	53.1 (± 10.9)	0.75
BMI (kg / m^2^)	21.1 (± 2.7)	21.4 (± 3.2)	21.1 (± 4.8)	0.94
HD duration (years)	8.6 (± 7.4)	9.5 (± 9.9)	8.2 (± 8.6)	0.79
Primary cause of end-stage renal disease (%)				0.49
Glomerulonephritis	35.0	35.0	21.4	
Diabetic nephropathy	25.0	37.5	40.5	
IgA nephropathy	15.0	2.5	4.8	
Polycystic kidney	2.5	2.5	4.8	
Nephrosclerosis	5.0	2.5	4.8	
Unknown	15.0	12.5	11.9	
Others	2.5	7.5	11.9	
Laboratory values				
Hemoglobin (g / dL)	12.7 (± 4.6)	10.9 (± 2.6)	11.8 (± 4.9)	0.17
Serum albumin (g / dL)	3.8 (± 0.3)	3.8 (± 0.3)	3.8 (± 0.2)	0.36
Comorbid conditions				
Diabetes mellitus (%)	30.0	47.5	52.4	0.10
Peripheral arterial disease (%)	30.0	45.0	54.8	0.09
Cardiac disease (%)	45.0	55.0	71.4	0.04
Cerebrovascular disease (%)	10.0	27.5	28.6	0.08
Peripheral neuropathy (%)	15.0	30.0	45.2	0.01
Orthopedic abnormality (%)	62.5	65.0	57.1	0.76
Joint disease (%)	42.5	45.0	40.5	0.92
History of fracture (%)	10.0	10.0	35.7	<0.01
Others (%)	32.5	32.5	23.8	0.61
The number of comorbidities	1.9 (± 1.3)	2.7 (± 1.3)	3.1 (± 1.3)	<0.001
Walking ability				
Maximum walking speed (m/min)	112.2 (± 8.4)	93.6 (± 5.4)	68.4 (± 14.4)	<0.001
Motor function				
Leg strength (%BW)	48.3 (± 12.1)	42.3 (± 11.9)	35.7 (±12.1)	<0.001
Lower extremity flexibility (degrees)	196.0 (± 27.3)	186.9 (± 30.7)	180.8 (± 37.3)	0.11
Standing balance (seconds)	41.0 (± 24.0)	32.8 (± 25.4)	16.4 (± 21.4)	<0.001

Date are presented as mean (± standard deviation) or number of subject. BMI: body mass index, HD: hemodialysis, BW: body weight. The cut-off values defining the maximum walking speed tertiles were <88.8 m/min, 88.8–106.2 m/min, and ≥106.2 m/min for men; and <80.4 m/min, 80.4–98.4 m/min, and ≥98.4 m/min for women.

### Univariate logistic regression analysis by tertiles of maximum walking speed

Univariate logistic regression analysis revealed that age (odds ratio [OR] = 1.83, 95% confidence interval [CI] = 1.15–2.91; P < 0.05), cardiac disease (OR = 2.50, 95% CI = 1.12–5.57; P < 0.05), peripheral neuropathy (OR = 2.85, 95% CI = 1.28–6.35; P < 0.05), history of fracture (OR = 5.00, 95% CI = 1.90–13.13; P < 0.01), number of comorbidities (OR = 4.03, 95% CI = 1.83–8.86; P < 0.01), leg strength (OR = 0.51, 95% CI = 0.35–0.74; P < 0.001), and standing balance (OR = 0.70, 95% CI = 0.59–0.83; P < 0.001) were significantly associated with the lowest tertile, compared to the middle and highest tertiles (Table 3).

**Table 3:** Univariable logistic regression analysis for the discrimination between the lowest tertile vs. the middle and highest tertiles of maximum walking speed.

Variable	Units of Increase	Lowest tertile vs.Middle and Highest tertiles		
		OR	(95% CI)	P value
Age (years)	10 years	1.83	(1.15–2.91)	0.01
Height (cm)	1 cm	0.96	(0.91–1.01)	0.09
BMI (kg / m^2^)	1 kg / m^2^	0.99	(0.89–1.10)	0.85
HD duration (years)	1 year	0.99	(0.95–1.03)	0.60
Hemoglobin (g / dL)	0.1 g / dL	1.00	(0.91–1.01)	0.96
Serum albumin (g / dL)	0.1 g / dL	0.46	(0.13–1.72)	0.25
Diabetes mellitus (presence)	1 presence	1.74	(0.82–3.70)	0.15
Peripheral arterial disease (presence)	1 presence	1.98	(0.93–4.22)	0.08
Cardiac disease (presence)	1 presence	2.50	(1.12–5.57)	0.03
Cerebrovascular disease (presence)	1 presence	1.73	(0.72–4.15)	0.22
Peripheral neuropathy (presence)	1 presence	2.85	(1.28–6.35)	0.01
Orthopedic abnormality (presence)	1 presence	0.76	(0.35–1.63)	0.48
Joint disease (presence)	1 presence	0.87	(0.41–1.87)	0.73
History of fracture (presence)	1 presence	5.00	(1.90–13.13)	<0.01
Others (presence)	1 presence	0.65	(0.28–1.52)	0.32
The number of comorbidities	1	4.03	(1.83–8.86)	<0.01
Leg strength (%BW)	10%BW	0.51	(0.35–0.74)	<0.001
Lower extremity flexibility (degrees)	1 degree	0.99	(0.98–1.00)	0.09
Standing balance (seconds)	10 seconds	0.70	(0.59–0.83)	<0.001

OR: odds ratio, CI: confidence interval, BMI: body mass index, HD: hemodialysis, BW: body weight.

The presence of diabetes mellitus (OR = 2.33, 95% CI = 1.05–5.21; P < 0.05), peripheral arterial disease (OR = 2.25, 95% CI = 1.01–5.04; P < 0.05), cerebrovascular disease (OR = 3.51, 95% CI = 1.12–10.97; P < 0.05), peripheral neuropathy (OR = 3.44, 95% CI = 1.30–9.14; P <0.05), number of comorbidities (OR = 4.00, 95% CI = 1.65–9.72; P < 0.01), leg strength (OR = 0.54, 95% CI = 0.38–0.76; P <0.001), and standing balance (OR = 0.77, 95% CI = 0.66–0.90; P <0.01) were significantly associated with the lowest and middle tertiles, compared to the highest tertile (Table 4).

**Table 4:** Univariable logistic regression analysis for the discrimination between the lowest and middle tertiles vs. the highest tertile of maximum walking speed.

Variable	Units of Increase	Lowest and Middle tertiles vs.Highest tertile		
		OR	(95% CI)	P value
Age (years)	10 years	1.51	(0.97–2.36)	0.07
Height (cm)	1 cm	0.98	(0.93–1.02)	0.31
BMI (kg / m^2^)	1 kg / m^2^	1.01	(0.91–1.12)	0.87
HD duration (years)	1 year	1.00	(0.96–1.05)	0.89
Hemoglobin (g / dL)	0.1 g / dL	0.93	(0.85–1.02)	0.12
Serum albumin (g / dL)	0.1 g / dL	0.39	(0.10–1.59)	0.19
Diabetes mellitus (presence)	1 presence	2.33	(1.05–5.21)	0.04
Peripheral arterial disease (presence)	1 presence	2.25	(1.01–5.04)	0.04
Cardiac disease (presence)	1 presence	2.12	(0.98–4.57)	0.06
Cerebrovascular disease (presence)	1 presence	3.51	(1.12–10.97)	0.03
Peripheral neuropathy (presence)	1 presence	3.44	(1.30–9.14)	0.01
Orthopedic abnormality (presence)	1 presence	0.94	(0.43–2.04)	0.87
Joint disease (presence)	1 presence	1.01	(0.47–2.16)	0.99
History of fracture (presence)	1 presence	2.71	(0.86–8.60)	0.09
Others (presence)	1 presence	0.81	(0.36–1.84)	0.61
The number of comorbidities	1	4.00	(1.65–9.72)	<0.01
Leg strength (%BW)	10%BW	0.54	(0.38–0.76)	<0.001
Lower extremity flexibility (degrees)	1 degree	0.99	(0.98–1.00)	0.06
Standing balance (seconds)	10 seconds	0.77	(0.66–0.90)	<0.01

OR: odds ratio, CI: confidence interval, BMI: body mass index, HD: hemodialysis, BW: body weight.

### Multivariate logistic regression analysis by tertiles of maximum walking speed

Stepwise multivariate logistic regression analysis revealed that the presence of cardiac disease (OR = 3.33, 95% CI = 1.26–8.83; P < 0.05), history of fracture (OR = 3.35, 95% CI = 1.08–10.38; P < 0.05), leg strength (OR = 0.62, 95% CI = 0.40–0.95; P < 0.05), and standing balance (OR = 0.76, 95% CI = 0.63–0.92; P < 0.01) were significantly associated with the lowest tertile, compared to the middle and highest tertiles after adjustment for confounders (Table 5).

**Table 5:** Stepwise multivariable logistic regression analysis for the discrimination between the lowest tertile vs. the middle and highest tertiles of maximum walking speed, and between the lowest and middle tertiles vs. the highest tertile of maximum walking speed.

Variable	Units of Increase	Lowest tertile vs. Middle and Highest tertiles			Lowest and Middle tertiles vs. Highest tertile		
		OR	(95% CI)	P value	OR	(95% CI)	P value
Cardiac disease (presence)	1 presence	3.33	(1.26–8.83)	0.02	2.84	(1.18–6.84)	0.02
History of fracture (presence)	1 presence	3.35	(1.08–10.38)	0.04	-	-	-
Leg strength (%BW)	10%BW	0.62	(0.40–0.95)	0.03	0.57	(0.39–0.82)	<0.01
Standing balance (seconds)	10 seconds	0.76	(0.63–0.92)	<0.01	0.81	(0.68–0.97)	0.02

Stepwise multivariable logistic regression analysis was performed based on the lowest tertile, and the lowest and middle tertiles as the dependent variable age, women, body mass index, hemodialysis duration, hemoglobin, serum albumin, diabetes mellitus, peripheral arterial disease, cardiac disease, cerebrovascular disease, peripheral neuropathy, history of fracture (spine, lower extremity), leg strength, lower extremity flexibility, standing balance as the independent variables. OR: odds ratio, CI: confidence interval, BW: body weight.

Cardiac disease (OR = 2.84, 95% CI = 1.18–6.84; P < 0.05), leg strength (OR = 0.57, 95% CI = 0.39–0.82; P < 0.01), and standing balance (OR = 0.81, 95% CI = 0.68–0.97; P < 0.05) were also significantly associated with the lowest and middle tertiles, compared to the highest tertile after adjustment for confounders (Table 5).

## Discussion

In the present cross-sectional study, MWS of hemodialysis patients varied widely, ranging from 40 m/min to 130 m/min in men and 30 m/min to 120 m/min in women (Fig 2). We thus categorized MWS into sex-specific tertiles to identify determinants of decreased walking speed, as clinical differences might exist in risk factors associated with slow walking speed by category. Multivariate logistic analysis revealed that the factors that independently discriminated the lowest and middle tertiles from the highest tertile were the presence of cardiac disease, decreased leg strength, and poor standing balance after adjustment for confounders. On the other hand, factors that independently discriminated the lowest tertile from the middle and highest tertiles were the presence of cardiac disease, history of fracture, decreased leg strength, and poor standing balance after adjustment for confounders. Common determinants for slow walking speed were cardiac comorbidities and decreased lower extremity motor function.

Ostir et al. reported that patients with a cardiac incident (e.g., myocardial infarction, chronic heart failure) were significantly more likely to have poorer lower extremity performance, with an approximately 5- to 8-fold greater degree of deterioration in MWS compared to those with no such incident [2]. Yamamoto et al. also reported that the walking speed of patients with their first acute myocardial infarction incident was approximately 70% of that of well-functioning people, regardless of age and sex [31]. These findings suggest that onset of a cardiac event leads to decreased walking speed. In the present study, the proportion of patients with cardiac disease was considerably high in all three walking speed tertiles (e.g., 45% in the highest tertile). These conditions may explain why the presence of cardiac disease could commonly help predict both the lowest and highest tertiles of walking speed from other categories.

Many studies have shown that leg strength and balance are closely and significantly associated with walking speed in both healthy and disabled adults [27, 32–34]. Bohannon reported that leg strength was significantly associated with walking speed in healthy adults aged 20–79 years [27]. Furthermore, Kutsuna et al. demonstrated that leg strength had significant effects on walking speed in stable hemodialysis patients, independent of clinical characteristics and physical activity levels [12]. Our previous study assessed physical functions, including leg strength and standing balance, in a healthy adult population from the same region targeted in the present study [31]. The present study found a decrease in physical functions, even in leg strength (mean, 48.3%), of patients in the highest walking speed tertile to approximately 80% of that of healthy adults reported in the previous study. Moreover, patients in the lowest walking speed tertile showed a decrease in leg strength to approximately 60% of that of healthy adults, the mean value of which (35.7%) was far below the cutoff value of 40%, which is required to be considered for ambulatory assistance or devices such as a cane or walker [35]. Since hemodialysis patients who required walking assistance were excluded from this study, most patients in the lowest tertile (i.e., slow walking speed) likely had greater difficulty walking. Yet, Tiedemann et al. reported that balance was significantly associated with walking speed, independent of other physiological factors including leg strength, in older community-dwelling people [32]. In this study, we used one-leg standing time to assess standing balance. One-leg standing time reflects the ability to maintain the center of pressure or gravity within a base of one-leg support, which is particularly important in walking since a gait cycle mainly consists of a single (one-leg) stance. One-leg standing time in the highest and lowest tertiles was approximately 80% and 30% of that of healthy older adults [31], respectively.

A history of fracture significantly and independently discriminated the lowest tertile from the middle and highest tertiles. The incidence of bone fracture, occurring most frequently in the hip, among Japanese hemodialysis patients is reportedly about five-fold higher than that among the general population [36]. Indeed, 35% of hemodialysis patients in the lowest walking speed tertile reported a history of fracture in the spine or lower extremities. In the present study, patients with a history of fracture in the spine or lower extremities within a year prior to entering this study were excluded, since the majority of fracture patients require at least several months of rehabilitation treatment (e.g., physical therapy) to recover the levels of daily activity and physical function equivalent to those exhibited before the fracture [37, 38]. One possible reason for the association between a history of fracture and decreased walking speed is the delay of more than one year in improvements of physical function following fracture in hemodialysis patients. Several studies have shown that improvements in physical function before a fracture, such as muscle strength, balance, joint flexibility, and walking speed, tend to be delayed compared to improvements in the independence of daily and major activities in patients with a lower extremity fracture [39–41]. Moreover, walking adaptability, which is defined as the ability to adjust walking to behavioral goals and environmental circumstances, tends to be lower in patients with frailty, falls, fall-related injuries (fracture), and fear of falling [41–43]. We also found that the most common cause of fracture according to medical records was accidental falls in patients in the lowest tertile. Therefore, walking inadaptability, such as fear of falling following a fracture, might have contributed to the marked decrease in walking speed in hemodialysis patients.

Contrary to our expectations, the presence of peripheral neuropathy and peripheral artery disease were not significant predictors of slow walking speed, independent of other clinical variables. A number of previous studies reported that peripheral neuropathy was closely associated with reduced leg strength and standing balance in patients with diabetes mellitus [44–48]. As mentioned above, leg strength and standing balance were directly and independently associated with decreased walking speed in hemodialysis patients [12, 49]. Univariate regression analysis revealed that peripheral neuropathy was a significant predictor for both the lowest and highest walking speed tertiles, suggesting that peripheral neuropathy might be indirectly associated with slow walking speed in hemodialysis patients. Patients with peripheral artery disease are more likely to have a restricted ability to walk long distances quickly due to their leg symptoms [50–52]. Peripheral artery disease was shown to be a significant predictor only in the lowest tertile of walking speed in our univariate analysis, possibly because patients with severe peripheral artery disease who required walking assistance were excluded from the present study.

This study has some limitations worth noting. First, our study might lack general applicability due to the small sample size. Although we categorized patients into sex-specific tertiles of walking speed to identify determinants of slow walking speed, more accurate results may be obtained by analyzing data for each sex separately. Second, this study had a cross-sectional design; a longitudinal study should be carried out in the future to investigate changes in walking speed, physical functions, and comorbidities in order to further examine determinants of decreased walking ability in hemodialysis patients. Third, We did not collect objective data on physical activity in this study. Several studies have reported that physical activity is closely related to walking ability. On the other hand, many reports suggest that walking speed influences daily physical activity [12, 53]. Accordingly, we did not include data regarding physical activity in our study to avoid confusion about the results (i.e., factors are associated with slow walking speed).

To the best of our knowledge, this is the first study to examine determinants of slow walking speed in ambulatory patients undergoing maintenance hemodialysis. Leg strength and standing balance, both predictors of decreased walking speed, have been reported to improve with adequate exercise training, regardless of age and degree of deterioration. Saito et al. reported that a two-month individualized home-based exercise improved physical fitness and abilities, including leg strength, in hemodialysis patients [54]. A recent study also showed that a cardiac rehabilitation program including balance training is more effective in improving walking speed after hospital discharge in patients with ischemic heart disease [55]. Furthermore, several studies have shown that walking inadaptability (e.g., fear of falling following a fracture) improved with a comprehensive rehabilitation program [56]. These findings suggest that the presence of cardiac disease, a history of fracture, and reduced physical function are very important preventive and predictive factors of decreased MWS in ambulatory hemodialysis patients.

## Supporting Information

S1 File All data. (PDF)
