# Supplementary material for: Determinants of Slow Walking Speed in Ambulatory Patients Undergoing Maintenance Hemodialysis
Source: PLoS One. 2016 Mar 28;11(3):e0151037. doi: 10.1371/journal.pone.0151037 (PMC4809595; doi:10.1371/journal.pone.0151037)
Supplement: S1 File — (PDF) [file pone.0151037.s001.pdf]

ID	Sex、0：men	Age(years)	Hight(cm)	Weight(kg)	BMI (kg / m2)	HD duration (years)
1	0	80.94	160.0	49.5	19.34	9.4
2	1	78.47	159.0	50.0	19.78	26.9
3	1	69.98	154.0	50.0	21.08	24.5
4	1	74.00	162.0	48.3	18.40	19.5
5	1	62.64	148.0	41.8	19.08	24.7
6	1	65.35	152.0	39.5	17.10	37.4
7	0	71.66	165.0	71.8	26.37	10.2
8	1	54.62	164.0	79.8	29.67	5.2
9	0	72.37	155.0	61.3	25.52	3.3
10	0	69.94	167.0	62.5	22.41	13.5
11	1	62.86	151.0	47.5	20.83	24.9
12	1	64.23	152.0	51.5	22.29	23.0
13	0	63.11	173.0	61.4	20.52	11.1
14	1	61.82	160.0	47.0	18.36	24.2
15	1	62.53	157.0	46.6	18.91	13.9
16	1	62.86	157.0	56.0	22.72	12.3
17	0	53.51	164.0	69.0	25.65	0.9
18	0	64.21	159.0	42.0	16.61	3.1
19	1	65.39	152.0	54.8	23.72	23.0
20	1	63.11	161.0	53.9	20.79	11.7
21	1	65.15	150.0	40.8	18.13	12.3
22	1	63.71	148.6	47.2	21.37	17.0
23	0	60.83	174.0	62.0	20.48	17.0
24	0	73.80	168.0	59.0	20.90	11.2
25	1	61.92	161.0	52.9	20.41	10.1
26	1	55.82	158.0	48.5	19.43	10.0
27	1	68.75	150.0	42.8	19.02	13.5
28	1	59.13	150.8	45.8	20.14	21.8
29	0	73.75	157.5	52.5	21.16	19.2
30	0	56.60	167.0	66.8	24.00	14.9
31	0	77.88	168.0	65.3	23.14	20.0
32	0	65.32	158.0	46.3	18.55	2.1

33	1	62.62	163.0	43.7	16.45	20.6
34	1	67.95	150.0	47.0	20.89	20.3
35	0	62.89	162.0	47.7	18.18	32.6
36	1	71.33	159.0	62.8	24.84	2.6
37	1	42.18	149.0	47.2	21.26	11.2
38	0	56.93	174.5	71.8	23.58	17.0
39	1	55.58	160.0	50.0	19.53	17.1
40	0	72.10	156.5	47.9	19.60	13.8
41	1	69.72	146.5	44.5	20.73	22.2
42	1	60.88	155.0	55.5	23.10	17.3
43	0	75.73	156.0	54.5	22.39	0.3
44	1	69.12	155.0	36.2	15.07	8.2
45	0	80.12	166.0	55.8	20.25	7.3
46	1	59.07	163.0	92.0	34.63	6.7
47	1	61.43	152.0	37.5	16.23	35.4
48	0	79.08	149.0	37.5	16.89	8.9
49	0	69.56	165.0	67.5	24.79	6.5
50	1	66.67	145.0	46.3	22.02	18.4
51	1	64.65	160.0	50.0	19.53	36.7
52	0	70.81	161.0	66.2	25.54	6.9
53	0	62.02	163.0	58.5	22.02	1.3
54	0	83.26	158.0	50.1	20.07	2.9
55	1	65.32	146.0	41.7	19.56	5.1
56	0	67.64	167.0	54.0	19.40	2.1
57	0	68.33	170.0	55.3	19.13	6.5
58	1	80.31	159.0	38.4	21.30	6.7
59	1	75.64	162.0	57.2	21.80	5.8
60	0	74.41	169.0	54.5	19.08	7.2
61	1	61.84	153.0	45.7	19.52	5.5
62	0	77.67	149.0	41.0	18.47	4.7
63	1	67.08	160.0	50.5	19.73	20.7
64	0	72.51	162.5	58.0	21.96	4.3
65	0	74.20	168.0	74.0	26.22	5.3
66	0	72.94	160.0	47.2	18.44	1.3

67	0	61.04	170.0	67.5	23.36	4.7
68	0	69.90	161.0	51.8	19.98	0.6
69	0	72.45	170.0	56.0	19.38	7.4
70	0	74.34	159.0	47.0	18.60	0.8
71	0	80.11	153.0	42.8	18.28	1.5
72	1	60.16	155.0	51.5	21.44	4.4
73	0	77.40	168.0	56.7	20.09	4.8
74	0	77.12	156.0	48.8	20.05	9.0
75	0	72.61	172.0	65.4	22.11	3.7
76	1	46.93	157.0	56.9	23.08	4.4
77	0	69.52	150.0	58.0	25.78	4.8
78	1	69.07	144.6	37.6	17.98	4.2
79	1	78.36	142.0	36.6	18.15	3.7
80	0	76.14	163.0	57.0	21.45	3.2
81	0	73.58	163.0	51.5	19.38	4.0
82	1	52.63	161.0	71.4	27.55	4.7
83	0	74.87	158.0	50.0	20.03	4.8
84	0	60.55	160.0	56.5	22.07	5.9
85	0	70.21	161.0	67.2	25.92	2.6
86	1	77.53	148.0	42.6	19.45	2.4
87	1	72.30	162.5	47.5	18.00	10.6
88	0	61.49	170.0	72.0	24.91	3.8
89	1	59.21	153.0	46.7	19.95	2.7
90	0	74.78	155.0	54.8	22.81	2.9
91	1	44.47	156.0	65.0	26.71	2.2
92	0	70.44	162.0	48.9	18.63	3.3
93	1	69.76	150.0	56.3	25.02	2.5
94	0	74.95	160.0	71.8	28.05	2.7
95	0	73.02	167.0	59.3	21.26	2.8
96	0	73.74	166.0	63.5	23.04	3.9
97	0	62.09	175.0	66.5	21.71	8.1
98	1	89.48	141.0	41.5	20.87	1.9
99	1	66.29	157.5	56.6	22.82	1.9
100	0	61.50	173.0	70.0	23.40	1.5

101	0	60.10	167.0	55.0	19.72	12.4
102	0	78.75	162.6	67.0	25.34	3.5
103	0	83.82	165.0	53.0	19.47	1.7
104	1	67.90	155.2	57.5	23.87	1.7
105	1	53.71	152.0	44.8	19.39	2.6
106	0	76.50	161.0	51.0	19.68	1.4
107	0	58.45	170.0	75.0	25.95	2.5
108	0	78.80	162.0	50.0	19.05	1.8
109	0	79.80	162.0	44.0	16.77	2.1
110	1	68.47	150.0	52.5	23.33	0.8
111	1	67.70	147.0	50.7	23.46	0.9
112	0	49.21	170.0	62.7	21.70	1.4
113	1	93.13	140.0	36.6	18.67	0.4
114	1	73.70	157.0	61.1	24.79	0.5
115	0	60.96	175.6	58.7	19.04	3.1
116	0	84.12	170.0	56.5	19.55	0.7
117	1	62.99	161.0	45.2	17.44	0.8
118	1	64.40	148.0	60.0	27.39	0.3
119	1	64.45	150.0	53.0	23.56	0.5
120	0	78.02	156.4	52.7	21.54	3.1
121	0	57.12	168.0	57.2	20.27	3.1
122	1	58.83	146.0	52.5	24.63	10.3

Primary cause of end-stage renal disease（1：Glomerulonephritis，2：Diabetic nephropathy，3：IgA nephropathy、4： Polycystic kidney、5： Polycystic kidney，6：Unknown、7: Others）
1
4
3
1
3
1
1
2
7
3
1
1
2
1
3
1
5
1
1
2
1
1
1
1
2
1
7
1
6
1
1
2

4
6
1
6
2
1
3
1
3
6
2
2
2
2
1
7
2
1
7
1
2
1
2
1
6
2
4
2
7
6
1
2
5
7

2
2
1
2
7
1
7
2
2
1
6
6
1
2
1
2
2
6
2
6
1
2
1
2
2
2
2
2
6
2
1
6
1
2

4
2
6
3
5
7
2
2
6
6
2
6
5
3
1
5
3
2
2
2
2
2

Comorbid conditions（0:None, 1:Diabetes mellitus, 2:Peripheral arterial disease, 3:Cardiac disease, 4:Cerebrovascular disease, 5:Peripheral neuropathy, 6:Orthopedic abnormality, 7:History of faucture, 8:Joint disease, 9:Others)	Hemoglobin (g / dL)
3	9.5
2,4,5,6,7,8,9	11.5
3,6,8,9	10.4
4,6,9	10.9
6,8	10.8
3,6,8,9	10.7
2,6,8,9	12.1
1,2,3,5,6,9	8.7
0	11.6
6,8	10.5
5,6,8,9	10.5
6,7,8,9	10.8
1,2,4,6,8	9.8
6,8	11.3
3,4,6,7,8,9	10.2
1,2,6,9	10.2
0	11.2
0	30.6
6,8,9	34.4
1,6,9	18.3
3	11.3
3,6,8,9	24.7
2,3,4,6,8,9	11.3
2,3,6,8,9	15.6
1,2,3,5,6,7,8,9	23.6
3,6,8	10.8
3,5,6,8	11.6
6,8,9	9.3
6,7,8	10.2
3,6,8,9	11.7
6,8	10.0
1,3,6,8	17.2

2,3,6,8,9	10.6
0	10.6
3,5,6,8	10.0
1,2,4,5,6,8	9.6
1,2,3	10.3
3	13.6
2,6,9	11.7
6,8	11.2
3,5,6,9	9.8
6,8,9	13.6
1,2,3	12.9
1,5,6,8	9.1
1,2,3,5,6,7,8	10.6
1,3,5,6,7,8	10.2
4,6,8	8.9
3,4,6,8	10.3
1,2,5,6,8	11.1
2,3,6,7,8	10.8
2,3,6,8	11.5
6,8,9	18.8
1,2,5	11.1
2,3,5	10.7
1,5,6,7	10.5
1,2,3,4,5	11.8
2,3	9.2
1,5,6,7,9	11.3
2,3,6,7,8,9	9.7
1,2,3,5,6,7,8	9.3
0	9.9
2,3,4	10.7
3,4,6,8	10.9
1,3,4,5	10.9
2,3,4	10.6
2,3,4,6,8	11.8

1,2,3,4,6,8,9	10.6
1,2,3,6,7,8	9.6
2,3,4,5,6,8	8.6
1,2,3,6,7	11.6
1,2,3,5	11.6
4,5	10.0
2,3	10.9
1,2,3,5	12.2
1,2,3,6,8	10.4
3,4	10.1
1,2,3	11.9
6,9	9.8
3,5,6,8	9.7
1,2,5	10.1
0	9.8
1,2,5,6,8	11.4
1,3,6,7	9.7
3,4,6,7,9	11.7
1	11.4
2,3,6,9	9.8
1,2,3,5,6,7,8,9	10.6
1,5	10.1
6,7	11.5
1,2,4	11.5
1,5,6,7,9	26.9
1,4,6,9	10.9
1,2,4	8.6
1,3,5,6,8	10.4
3	11.6
1,2,3,5	11.0
2,3,4	23.7
3	10.1
0	11.1
1,5	9.4

1,3	11.4
1,2,3	10.8
1,3,5,6,7	9.6
3,6,8,9	9.4
6,9	10.3
2,3	10.6
1,3,6,9	11.9
1,2,3,6,7	9.8
3,6,7	10.8
6,7	10.1
1,2,4,6,9	10.4
1,3	23.5
1,4	11.4
1,2,3,5	10.3
2,3,6,8	17.8
2,3	11.8
6,8	10.6
1,5	8.8
1,2,5	9.5
1,2,3,4,5	10.9
1,2,3	11.6
1,2,3,4	10.0

Serum albumin (g / dL)	Leg strength (%BW)	Standing balance (seconds)	Lower extremity flexibility (degrees)
4.0	47.68	60.0	120
3.8	18.25	4.3	190
3.5	37.55	60.0	200
4.1	27.50	60.0	205
3.6	54.43	60.0	240
3.6	47.40	60.0	210
3.7	56.96	60.0	200
3.8	15.35	1.1	125
3.8	41.47	31.0	185
3.8	74.00	60.0	170
3.8	41.16	60.0	205
3.7	42.91	60.0	195
3.8	38.03	3.2	180
4.0	40.11	60.0	190
3.6	35.41	60.0	190
4.0	48.13	43.6	165
4.1	35.94	56.4	185
3.9	46.79	60.0	190
4.1	33.48	60.0	180
4.0	55.57	21.3	190
4.1	52.82	60.0	210
4.1	40.68	10.0	215
3.8	37.10	4.9	185
3.6	76.61	14.0	205
4.0	32.80	46.0	235
4.4	38.04	60.0	225
3.3	34.16	60.0	175
2.8	48.34	60.0	165
3.6	45.14	60.0	200
4.1	41.30	57.7	250
3.7	33.23	24.4	170
3.7	74.19	30.7	170

3.7	30.90	24.2	215
3.8	53.09	60.0	185
3.3	27.57	60.0	130
3.9	19.22	2.2	160
4.0	55.30	5.1	245
3.7	44.08	60.0	210
3.9	55.50	60.0	200
3.9	42.80	27.0	190
3.6	23.82	0.0	170
4.3	58.56	60.0	215
3.8	44.50	11.3	175
4.0	48.63	2.3	225
3.3	36.38	1.5	155
3.6	18.53	1.0	70
3.8	30.80	60.0	205
3.5	72.13	60.0	200
3.5	47.56	19.7	175
3.9	30.13	3.8	165
3.8	41.99	16.5	220
3.6	53.40	20.1	190
3.7	18.46	1.3	150
4.2	47.01	2.4	175
3.7	26.02	10.1	195
3.8	42.92	5.8	125
3.4	58.95	60.0	180
3.7	26.41	0.5	185
3.6	20.80	5.5	155
3.7	38.29	1.7	120
3.9	46.72	2.5	210
3.9	46.71	60.0	170
3.7	31.39	1.2	210
4.1	39.05	10.8	160
3.9	32.23	53.8	150
4.1	55.51	60.0	235

4.1	46.44	3.3	205
4.0	46.72	6.2	160
2.9	33.75	7.5	230
3.9	40.11	24.0	195
4.1	39.95	33.1	190
3.5	22.23	5.5	200
4.0	61.20	35.7	220
3.6	56.97	29.4	165
4.2	37.54	60.0	205
4.1	40.32	60.0	235
3.9	67.93	7.8	200
4.1	48.65	60.0	200
4.2	21.49	2.4	170
3.9	48.77	3.3	180
3.6	62.72	2.1	195
3.7	29.34	4.7	180
3.8	48.50	10.8	275
4.0	39.73	60.0	195
4.0	51.35	10.1	190
3.8	41.55	60.0	205
3.5	10.21	7.7	145
4.0	30.14	2.2	200
4.4	52.46	60.0	225
3.5	47.35	3.2	180
3.6	26.75	1.1	220
4.0	42.43	11.1	160
3.6	47.47	3.8	145
4.0	32.24	4.8	205
4.1	36.93	54.2	200
3.5	38.11	1.2	120
3.0	50.45	60.0	155
4.0	39.40	8.7	210
3.8	33.36	60.0	200
4.3	39.70	25.1	205

4.0	53.99	60.0	210
3.9	55.75	14.7	120
3.4	35.14	8.3	155
3.9	73.83	60.0	135
3.8	47.18	60.0	225
3.8	42.55	6.3	155
3.7	47.20	10.7	185
3.6	42.10	0.7	175
3.9	51.25	9.3	215
3.3	32.19	60.0	165
3.7	30.78	2.3	165
4.0	38.36	60.0	170
3.4	42.00	0.0	215
3.2	29.62	8.8	200
3.9	53.67	60.0	225
3.8	58.76	19.8	230
3.8	31.86	60.0	260
3.2	24.58	0.3	200
3.7	28.40	7.6	170
3.7	32.55	1.7	150
4.0	50.86	21.7	225
4.2	56.57	60.0	160

Maximum walking speed(m/min)
114.29
59.35
98.20
98.40
109.89
92.74
105.26
91.19
89.82
114.94
93.75
89.69
89.82
118.58
118.20
97.24
113.64
105.82
75.47
106.95
95.85
100.84
64.86
120.00
74.35
127.66
93.75
92.74
83.10
114.00
83.33
112.78

86.33
102.39
96.46
83.33
71.68
89.82
119.52
93.60
48.00
122.70
114.29
93.02
113.21
80.21
94.34
81.08
106.76
59.41
58.42
106.76
76.05
119.76
86.96
54.25
114.07
35.78
46.20
47.21
55.76
86.83
56.02
95.54
69.04
131.87

104.90
88.24
96.93
66.01
73.44
79.79
102.21
88.89
94.94
96.15
122.95
99.67
46.77
111.32
112.57
103.45
75.00
88.24
93.90
86.33
55.20
89.55
113.64
101.87
52.54
92.74
72.12
75.47
80.97
83.92
116.05
62.63
108.11
106.80

121.21
104.90
89.15
97.24
106.76
85.59
113.21
60.67
89.69
100.50
94.49
90.63
80.40
76.14
97.24
95.24
99.67
45.91
84.75
75.28
120.72
92.31
